# Impact in Italy of research in pediatrics

**DOI:** 10.1186/1824-7288-40-S1-A45

**Published:** 2014-08-11

**Authors:** Generoso Andria

**Affiliations:** 1 Department of Translational Medicine – Section of Pediatrics, University of Naples Federico II, Naples, Italy

## 

We examined several parameters related to pediatric research in Italy over the past few years,. We distinguished the research carried out by the scientific community as a whole on topics of pediatric interest from the research carried out by pediatricians. We considered only studies in which an Italian institution played a leading role and excluded studies with Italian collaborators, but coordinated by foreign researchers. Data relating to Italian pediatric research were retrieved from national and international sources and databases, such as PubMed, and compared with those obtained with the same methodology for the following European countries: France, Germany, United Kingdom, the Netherlands, Spain, Greece . The results obtained were then corrected for the gross domestic product at purchasing power parity per capita of any nation, which takes into account the number of inhabitants and the purchasing power of resources related to that country. From these data we can tentatively conclude that the Italian pediatric research, despite fewer resources invested, is at the same level, of European countries with comparable population and much higher investments devoted to scientific research. Another “Italian miracle” or a case of “submerged economy”, even in research?

